# Supplementary material for: Benchmarking Measures of Network Influence
Source: Sci Rep. 2016 Sep 27;6:34052. doi: 10.1038/srep34052 (PMC5037445; doi:10.1038/srep34052)
Supplement: Supplementary Information [file srep34052-s1.pdf]

Supplementary Material for:
Benchmarking Measures of Network Influence

Aaron Bramson[1,2,3,*] **and Benjamin Vandermarliere**[2,4]

[1]Riken Brain Science Institute, Laboratory for Symbolic Cognitive Development, Wako, 351-0198, Japan
[2]Ghent University, Department of General Economics, Ghent, 9000, Belgium
[3]University of North Carolina at Charlotte, Department of Software and Information Systems, Charlotte, 28223, USA
[4]Ghent University, Department of Physics and Astronomy, Ghent, 9000, Belgium
[*]bramson@brain.riken.jp

Content Overview

This appendix presents details of the results that may be of interest to specialist looking to reproduce the technique and/or for a better understand the nuances of the approach. Our python code for running the simulations and calculated the measure values is available upon serious, verifiable request. The appendix material is presented in the following sections.

Model Scenarios and Infection Sizes: Includes a 3D histogram with a row for each scenario showing the frequency of infections of each size. We also have a set of twelve 3D histograms (one for each scenario) with a row for each of the 25 skeletons showing the disease variation resulting from network structure; however, in consideration of space and the real focus of this paper they are excluded (available upon request). We also provide a table of the mean and standard deviations of the raw magnitudes for each scenario. When excluding the duds the distributions approximate normal distributions, but the large numbers of duds make the normal approximation inappropriate and it is *not* the case that the disease results follow any single distribution with the mean and standard deviations in the table. The mean and standard deviation do, however, capture the relative all-things-considered expected infection sizes for each scenario.

Correlations between Agent-Initialized Magnitude and TKO Measures: The Pearson and Spearman correlation coefficients between (a) the disease magnitude reached when agent i is the initial agent and (b) four different versions of the TKO aggregated across time for agent i. The correlations are performed separately for each scenario between the lists of values for all 200 agents combined across all 25 network skeletons. With the highest scores near 0.50 and most much lower, the result is that measuring an agent's impact using the super-spreader approach alone is not accurate in capturing an agent's actual influence compared to TKO.

Comparisons of Network Measures to TKO scores: This section starts with one page of further methodological description, especially about the flattened observed interaction dynamics networks. Following that are eight pages of table triplets each showing the Pearson, Spearman, and Top Ten comparisons between each of five common network centrality measures. Each of the four TKO variation has its one page of tables for both the base and the flattened networks. Because the base and unweighted flattened networks are nearly identical, so are the correlations. The weighted versions of the flattened measure are excluded in consideration of the space to describe them in light of the result that they also do not significantly covary with any TKO measures.

Results Details

Model Scenarios and Infection Sizes

As seen in Appendix Table 1 both network types show high variation in magnitude depending on the initial agent; however, when aggregated across the 25 implementations of each network type they reveal similar magnitude profiles. There are a large number of runs in which the disease fewer than 50 agents-times (what we call "duds", see Appendix Figure 1) and although these outcomes drag the mean magnitude down and raise the variance, for our purposes there is no benefit in separating out the duds and, for example, testing the remaining infections for matches to known distributions.

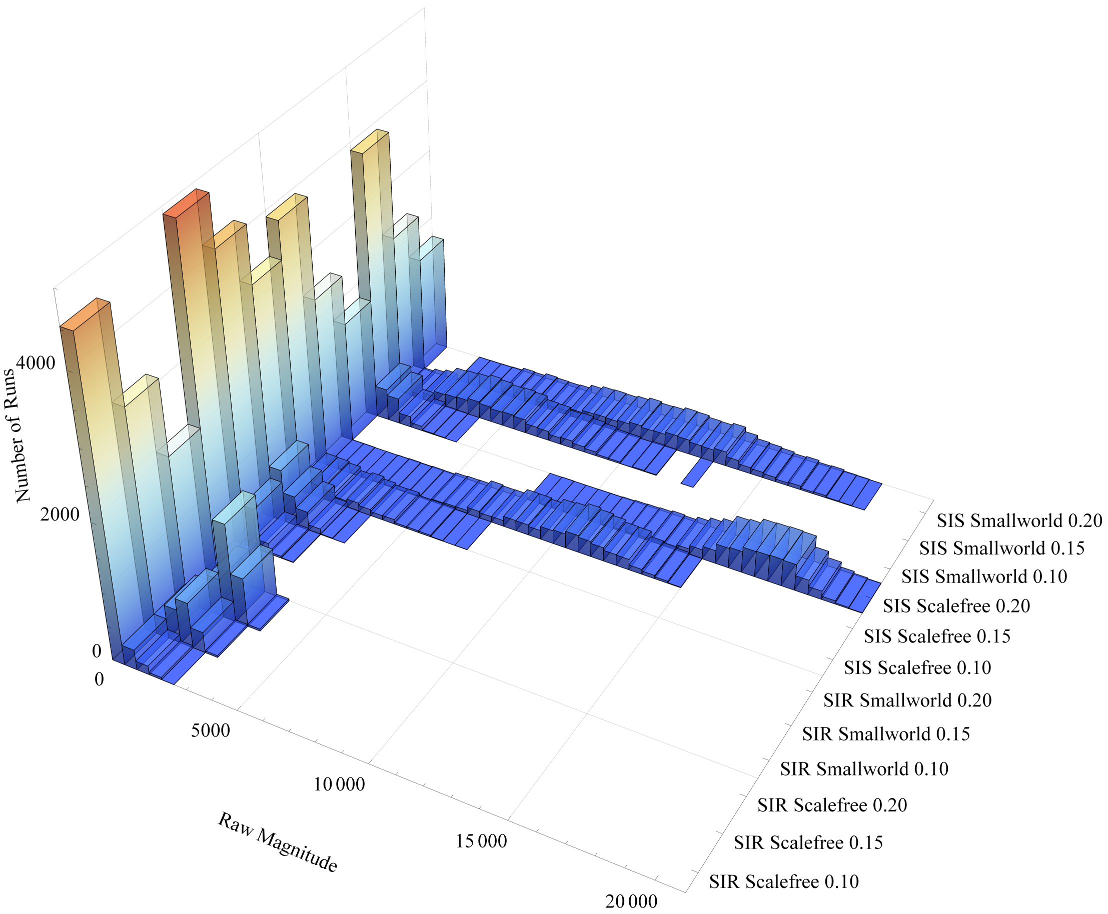

Figure 1. Results histogram of infection spread in terms of the number of temporal nodes infected (raw magnitude) across 5000 runs for each scenario (one run initialized at each of 200 agents for each of the 25 base network implementations). Notice that a very large proportion of runs are "duds" in which the infection fails to spread beyond 50 temporal nodes. The SIS models naturally have greater magnitude values due to reinfection. These dynamics are typical of SIR and SIS models with similar parameters.

Infection Type	Network Type	Infection Probability	Mean Magnitude	Magnitude StDev	Percent Duds
SIR	Scale Free	0.10	143.352	288.549	0.625
SIR	Scale Free	0.15	584.744	774.628	0.482
SIR	Scale Free	0.20	1296.44	1142.24	0.380
SIR	Small World	0.10	88.9266	131.743	0.584
SIR	Small World	0.15	227.321	324.207	0.457
SIR	Small World	0.20	445.033	559.017	0.352
SIS	Scale Free	0.10	548.746	1155.19	0.593
SIS	Scale Free	0.15	5003.03	5237.44	0.445
SIS	Scale Free	0.20	10800.6	8150.76	0.344
SIS	Small World	0.10	308.734	536.106	0.557
SIS	Small World	0.15	2526.97	2839.26	0.433
SIS	Small World	0.20	7036.79	5623.18	0.333

Table 1. Results summary of infection spread for each model variation. Each row aggregates 5000 runs (one run initialized at each of 200 agents for each of the 25 base network implementations). Duds are defined as runs in which the raw magnitude is fewer than 50 agent-times.

Correlations between Agent-Initialized Magnitude and TKO Measures

Pearson Correlations of Agent-Initialized Magnitude and TKO Measures.

Disease Type	Network Type	InfectionRate	MaxProportion	MaxDeltaFraction	AveProportion	AveDeltaFraction
SIR	scalefree	0.10	0.403	0.405	0.288	0.292
SIR	scalefree	0.15	0.067	0.246	0.064	0.157
SIR	scalefree	0.20	0.046	0.219	0.078	0.158
SIR	smallworld	0.10	0.494	0.472	0.366	0.364
SIR	smallworld	0.15	0.043	0.265	0.077	0.189
SIR	smallworld	0.20	0.03	0.192	0.015	0.119
SIS	scalefree	0.10	0.347	0.376	0.268	0.282
SIS	scalefree	0.15	0.057	0.248	0.084	0.153
SIS	scalefree	0.20	0.045	0.234	0.059	0.108
SIS	smallworld	0.10	0.404	0.418	0.353	0.371
SIS	smallworld	0.15	0.024	0.201	0.045	0.15
SIS	smallworld	0.20	0.05	0.19	0.042	0.107

Table 2. The mean Pearson correlations coefficients of (a) the disease magnitude given an agent is the initially infected agent and (b) the TKO score for that agent. The on-average low correlations imply that using the disease spread based on initial infection is a poor measure of influence. Furthermore, the correlations are nearly always worse with increasing infection rates (and hence increasing magnitudes and fewer dud runs) implying that much of the ability to match TKO relies on the cases in which both scores are near zero.

Spearman Correlations of Agent-Initialized Magnitude and TKO Measures.

Disease Type	Network Type	InfectionRate	MaxProportion	MaxDeltaFraction	AveProportion	AveDeltaFraction
SIR	scalefree	0.10	0.402	0.305	0.388	0.309
SIR	scalefree	0.15	0.063	0.221	0.106	0.194
SIR	scalefree	0.20	0.028	0.203	0.067	0.169
SIR	smallworld	0.10	0.517	0.42	0.461	0.404
SIR	smallworld	0.15	0.067	0.293	0.149	0.27
SIR	smallworld	0.20	0.038	0.223	0.058	0.172
SIS	scalefree	0.10	0.297	0.253	0.277	0.236
SIS	scalefree	0.15	0.049	0.198	0.081	0.136
SIS	scalefree	0.20	0.037	0.185	0.028	0.07
SIS	smallworld	0.10	0.335	0.281	0.296	0.256
SIS	smallworld	0.15	0.026	0.191	0.08	0.161
SIS	smallworld	0.20	0.045	0.188	0.045	0.114

Table 3. The mean Spearman Rank correlation coefficients (rho) of (a) the disease magnitude given an agent is the initially infected agent and (b) the TKO score for that agent. The correlations reveal similar values and a similar pattern to the Pearson correlations, reinforcing that using the disease spread based on initial infection is a poor measure of influence.

Correlations between Agent-Initialized Epidemic Probability and TKO Measures

Pearson Correlations of Agent-Initialized Epidemic Probability and TKO Measures.

Disease Type	Network Type	InfectionRate	MaxProportion	MaxDeltaFraction	AveProportion	AveDeltaFraction
SIR	scalefree	0.10	0.228	0.176	0.183	0.137
SIR	scalefree	0.15	0.07	0.16	0.082	0.105
SIR	scalefree	0.20	0.048	0.196	0.083	0.144
SIR	smallworld	0.10	0.323	0.221	0.254	0.183
SIR	smallworld	0.15	0.075	0.175	0.111	0.134
SIR	smallworld	0.20	0.046	0.078	0.047	0.056
SIS	scalefree	0.10	0.171	0.155	0.148	0.126
SIS	scalefree	0.15	0.056	0.194	0.096	0.127
SIS	scalefree	0.20	0.043	0.219	0.067	0.108
SIS	smallworld	0.10	0.172	0.137	0.158	0.129
SIS	smallworld	0.15	0.036	0.116	0.076	0.103
SIS	smallworld	0.20	0.042	0.136	0.054	0.087

Table 4. The mean Pearson correlations coefficients of (a) the proportion of runs infecting more than 50 temporal nodes (not "duds") given an agent is the initially infected agent and (b) the TKO score for that agent. The on-average low correlations imply that using the disease spread based on initial infection is a poor measure of influence. Furthermore, the correlations are nearly always worse with increasing infection rates (and hence increasing magnitudes and fewer dud runs) implying that much of the ability to match TKO relies on the cases in which both scores are near zero.

Spearman Correlations of Agent-Initialized Epidemic Probability and TKO Measures.

Disease Type	Network Type	InfectionRate	MaxProportion	MaxDeltaFraction	AveProportion	AveDeltaFraction
SIR	scalefree	0.10	0.409	0.306	0.398	0.314
SIR	scalefree	0.15	0.07	0.198	0.107	0.18
SIR	scalefree	0.20	0.033	0.209	0.07	0.174
SIR	smallworld	0.10	0.518	0.41	0.462	0.404
SIR	smallworld	0.15	0.079	0.262	0.15	0.249
SIR	smallworld	0.20	0.054	0.151	0.074	0.121
SIS	scalefree	0.10	0.273	0.217	0.267	0.211
SIS	scalefree	0.15	0.052	0.186	0.092	0.137
SIS	scalefree	0.20	0.035	0.204	0.052	0.099
SIS	smallworld	0.10	0.282	0.205	0.252	0.191
SIS	smallworld	0.15	0.025	0.142	0.087	0.128
SIS	smallworld	0.20	0.037	0.151	0.059	0.098

Table 5. The mean Spearman Rank correlation coefficients (rho) of (a) the proportion of runs infecting more than 50 temporal nodes (not "duds") given an agent is the initially infected agent and (b) the TKO score for that agent. The correlations reveal similar values and a similar pattern to the Pearson correlations, reinforcing that using the disease spread based on initial infection is a poor measure of influence.

Comparisons of Network Measures to TKO scores

The following twelve sets of three data tables present the results of determining how well common network centrality measures capture agent influence. Although the main result is that none of the network measures successfully capture/predict agent influence as measured by four versions of TKO in any scenario, the specific changes in the data reveal patterns – and those patterns may point to improved measures.

Although the paper focuses on the base network analysis, we also analyzed the network generated by flattening the observed interactions. We record who interacts with whom over time in the temporal network skeleton, then we flatten this skeleton to achieve both a weighted by interaction frequency and an unweighted flat network representation. If the model runs long enough the observed interactions converge to the base network of potential interactions, but in many applications the flattened network is observable/derivable from data while the base network is unknown and/or theoretical. In our simulations, because the probability that a given link is active in a time step is $\propto 1/k$, k is low (typically single digit except a few agents in the scale free networks), and there are 200 time steps, the base and unweighted flattened graphs are nearly identical.

Because for each base network we generate the skeleton including all transition and interaction probabilities, the empirically derived flattened network connections are always the same for each run of the same skeleton (i.e., starting from each agent). In the current model the infection state does not alter the interaction probability. If it did, then the observed transitions would vary from run to run even using the same network skeleton because what is stored in the skeleton is a set of draws from probability distributions rather than a fixed interaction structure. If, for example, being infectious reduced the probability of interaction, then the probability stored in the skeleton would be compared to a different interaction threshold and thus could alter which interactions occur. However, using the same skeletons for multiple runs of different dynamics on the same structure at least satisfies the Markov condition for these simulations, which is not maintained when running the dynamics independently for each initial agent run.

Flattened graphs are potentially better at tracking influence because they allow one to create weighted networks from the observed interaction frequencies. However, in our experiments the correlation valued between TKO and the weighted network centrality measures were no better, although they were slightly different. For this reason and considerations of space we have excluded them from this paper.

Maximum TKO and Base Interaction Network

Pearson Correlations of Centrality Measures and Maximum TKO on Base Network

Disease Type	Network Type	InfectionRate	Degree	Closeness	Betweenness	Eigenvector	Katz	Accessibility	Expected Force
SIR	scalefree	0.10	0.123	0.096	0.096	0.089	0.069	0.075	0.112
SIR	scalefree	0.15	0.147	0.113	0.127	0.115	0.099	0.086	0.131
SIR	scalefree	0.20	0.144	0.114	0.119	0.114	0.099	0.094	0.133
SIR	smallworld	0.10	0.007	0.065	0.038	0.	−0.005	0.061	0.004
SIR	smallworld	0.15	0.059	0.085	0.11	0.085	0.075	0.112	0.062
SIR	smallworld	0.20	0.083	0.179	0.14	0.032	0.074	0.172	0.079
SIS	scalefree	0.10	0.138	0.096	0.119	0.099	0.078	0.073	0.114
SIS	scalefree	0.15	0.118	0.082	0.097	0.087	0.073	0.056	0.099
SIS	scalefree	0.20	0.095	0.077	0.076	0.073	0.06	0.055	0.087
SIS	smallworld	0.10	0.022	0.08	0.035	−0.013	0.005	0.06	0.018
SIS	smallworld	0.15	0.064	0.116	0.114	0.027	0.055	0.12	0.062
SIS	smallworld	0.20	0.118	0.137	0.23	0.066	0.134	0.205	0.119

Spearman Correlations of Centrality Measures and Maximum TKO on Base Network

Disease Type	Network Type	InfectionRate	Degree	Closeness	Betweenness	Eigenvector	Katz	Accessibility	Expected Force
SIR	scalefree	0.10	0.192	0.104	0.189	0.093	0.061	0.072	0.131
SIR	scalefree	0.15	0.199	0.107	0.178	0.105	0.074	0.07	0.136
SIR	scalefree	0.20	0.212	0.122	0.208	0.116	0.088	0.083	0.149
SIR	smallworld	0.10	0.012	0.028	0.038	0.031	0.011	0.043	0.002
SIR	smallworld	0.15	0.056	0.093	0.091	0.062	0.046	0.088	0.04
SIR	smallworld	0.20	0.088	0.172	0.102	0.074	0.086	0.15	0.068
SIS	scalefree	0.10	0.19	0.092	0.19	0.084	0.052	0.056	0.119
SIS	scalefree	0.15	0.188	0.084	0.174	0.083	0.055	0.041	0.114
SIS	scalefree	0.20	0.194	0.086	0.19	0.08	0.048	0.034	0.116
SIS	smallworld	0.10	0.016	0.044	0.028	−0.004	−0.006	0.054	−0.008
SIS	smallworld	0.15	0.083	0.159	0.146	0.034	0.051	0.15	0.049
SIS	smallworld	0.20	0.114	0.169	0.203	0.096	0.14	0.207	0.13

Top Ten Overlap of Centrality Measures and Maximum TKO on Base Network

Disease Type	Network Type	InfectionRate	Degree	Closeness	Betweenness	Eigenvector	Katz	Accessibility	Expected Force
SIR	scalefree	0.10	0.08	0.1	0.076	0.092	0.088	0.084	0.092
SIR	scalefree	0.15	0.104	0.108	0.108	0.1	0.096	0.104	0.104
SIR	scalefree	0.20	0.084	0.08	0.084	0.08	0.084	0.08	0.08
SIR	smallworld	0.10	0.044	0.056	0.048	0.028	0.04	0.068	0.06
SIR	smallworld	0.15	0.064	0.1	0.112	0.072	0.068	0.076	0.06
SIR	smallworld	0.20	0.108	0.116	0.116	0.044	0.08	0.1	0.088
SIS	scalefree	0.10	0.112	0.1	0.108	0.096	0.1	0.104	0.112
SIS	scalefree	0.15	0.08	0.08	0.076	0.076	0.072	0.064	0.072
SIS	scalefree	0.20	0.068	0.064	0.064	0.048	0.052	0.06	0.06
SIS	smallworld	0.10	0.072	0.064	0.084	0.068	0.076	0.06	0.088
SIS	smallworld	0.15	0.092	0.092	0.104	0.052	0.084	0.128	0.096
SIS	smallworld	0.20	0.124	0.14	0.164	0.048	0.16	0.16	0.156

Table 6. The Pearson and Spearman correlations as well as the average percent of matching Top Ten agents between the maximum proportional TKO score with each of seven base network agent centrality scores.

Maximum TKO and Flattened Interaction Network - Unweighted

Pearson Correlations of Unweighted Centrality Measures and Maximum TKO on Flattened Network

Disease Type	Network Type	InfectionRate	Degree	Closeness	Betweenness	Eigenvector	Katz	Accessibility	Expected Force
SIR	scalefree	0.10	0.123	0.094	0.097	0.071	−0.015	0.074	0.112
SIR	scalefree	0.15	0.147	0.11	0.128	0.054	0.014	0.085	0.131
SIR	scalefree	0.20	0.144	0.115	0.12	0.035	0.003	0.094	0.133
SIR	smallworld	0.10	0.007	0.065	0.038	−0.033	−0.008	0.061	0.004
SIR	smallworld	0.15	0.059	0.085	0.11	0.042	−0.001	0.112	0.062
SIR	smallworld	0.20	0.083	0.179	0.14	−0.021	0.016	0.172	0.079
SIS	scalefree	0.10	0.139	0.094	0.119	0.075	0.004	0.072	0.114
SIS	scalefree	0.15	0.118	0.079	0.097	0.07	0.023	0.054	0.099
SIS	scalefree	0.20	0.096	0.078	0.077	0.028	0.014	0.056	0.088
SIS	smallworld	0.10	0.022	0.08	0.035	−0.002	−0.006	0.06	0.018
SIS	smallworld	0.15	0.064	0.116	0.114	0.001	0.011	0.12	0.062
SIS	smallworld	0.20	0.118	0.137	0.23	−0.036	0.024	0.205	0.119

Spearman Correlations of Unweighted Centrality Measures and Maximum TKO on Flattened Network

Disease Type	Network Type	InfectionRate	Degree	Closeness	Betweenness	Eigenvector	Katz	Accessibility	Expected Force
SIR	scalefree	0.10	0.192	0.104	0.189	0.068	−0.018	0.072	0.131
SIR	scalefree	0.15	0.199	0.107	0.181	0.067	0.024	0.07	0.136
SIR	scalefree	0.20	0.212	0.122	0.207	0.039	0.003	0.083	0.149
SIR	smallworld	0.10	0.012	0.028	0.038	−0.053	−0.003	0.043	0.002
SIR	smallworld	0.15	0.056	0.093	0.091	0.058	−0.002	0.089	0.04
SIR	smallworld	0.20	0.088	0.172	0.102	0.025	0.015	0.15	0.068
SIS	scalefree	0.10	0.19	0.092	0.191	0.059	−0.001	0.057	0.12
SIS	scalefree	0.15	0.188	0.082	0.176	0.064	0.039	0.041	0.113
SIS	scalefree	0.20	0.194	0.086	0.19	0.026	0.015	0.035	0.117
SIS	smallworld	0.10	0.016	0.044	0.028	−0.019	−0.014	0.053	−0.008
SIS	smallworld	0.15	0.083	0.159	0.146	0.043	0.007	0.15	0.049
SIS	smallworld	0.20	0.114	0.169	0.203	0.012	0.	0.206	0.13

Top Ten Overlap of Unweighted Centrality Measures and Maximum TKO on Flattened Network

Disease Type	Network Type	InfectionRate	Degree	Closeness	Betweenness	Eigenvector	Katz	Accessibility	Expected Force
SIR	scalefree	0.10	0.084	0.096	0.076	0.1	0.048	0.096	0.092
SIR	scalefree	0.15	0.1	0.1	0.108	0.052	0.096	0.104	0.1
SIR	scalefree	0.20	0.084	0.076	0.084	0.044	0.08	0.076	0.08
SIR	smallworld	0.10	0.044	0.056	0.048	0.044	0.044	0.068	0.06
SIR	smallworld	0.15	0.064	0.1	0.112	0.068	0.024	0.08	0.06
SIR	smallworld	0.20	0.108	0.116	0.116	0.06	0.06	0.1	0.088
SIS	scalefree	0.10	0.116	0.1	0.108	0.112	0.044	0.108	0.112
SIS	scalefree	0.15	0.072	0.064	0.076	0.1	0.068	0.056	0.068
SIS	scalefree	0.20	0.068	0.064	0.068	0.052	0.08	0.06	0.06
SIS	smallworld	0.10	0.072	0.064	0.084	0.084	0.04	0.06	0.088
SIS	smallworld	0.15	0.092	0.092	0.104	0.044	0.04	0.132	0.096
SIS	smallworld	0.20	0.124	0.14	0.164	0.04	0.064	0.16	0.156

Table 7. The Pearson and Spearman correlations as well as the average percent of matching Top Ten agents between the maximum proportional TKO score with each of seven flattened observed interaction network agent centrality scores.

Maximum Delta Fraction TKO and Base Interaction Network

Pearson Correlations of Centrality Measures and Maximum Delta TKO on Base Network

Disease Type	Network Type	InfectionRate	Degree	Closeness	Betweenness	Eigenvector	Katz	Accessibility	Expected Force
SIR	scalefree	0.10	0.103	0.083	0.08	0.075	0.058	0.066	0.094
SIR	scalefree	0.15	0.167	0.132	0.151	0.139	0.125	0.111	0.15
SIR	scalefree	0.20	0.153	0.121	0.128	0.12	0.104	0.099	0.141
SIR	smallworld	0.10	0.005	0.087	0.047	−0.007	−0.01	0.074	0.001
SIR	smallworld	0.15	0.086	0.184	0.147	0.078	0.09	0.198	0.086
SIR	smallworld	0.20	0.111	0.207	0.168	0.041	0.102	0.203	0.109
SIS	scalefree	0.10	0.118	0.085	0.102	0.089	0.073	0.069	0.099
SIS	scalefree	0.15	0.138	0.101	0.116	0.106	0.092	0.074	0.119
SIS	scalefree	0.20	0.114	0.09	0.094	0.087	0.073	0.065	0.102
SIS	smallworld	0.10	0.018	0.116	0.045	−0.01	0.001	0.079	0.014
SIS	smallworld	0.15	0.081	0.236	0.158	0.045	0.073	0.21	0.079
SIS	smallworld	0.20	0.141	0.201	0.269	0.074	0.153	0.26	0.142

Spearman Correlations of Centrality Measures and Maximum Delta TKO on Base Network

Disease Type	Network Type	InfectionRate	Degree	Closeness	Betweenness	Eigenvector	Katz	Accessibility	Expected Force
SIR	scalefree	0.10	0.192	0.105	0.19	0.093	0.058	0.07	0.13
SIR	scalefree	0.15	0.222	0.122	0.214	0.12	0.09	0.088	0.155
SIR	scalefree	0.20	0.232	0.134	0.226	0.125	0.093	0.096	0.165
SIR	smallworld	0.10	0.016	0.055	0.049	0.035	0.009	0.069	−0.002
SIR	smallworld	0.15	0.061	0.177	0.097	0.077	0.063	0.155	0.052
SIR	smallworld	0.20	0.099	0.241	0.141	0.074	0.087	0.204	0.071
SIS	scalefree	0.10	0.184	0.089	0.187	0.079	0.047	0.053	0.114
SIS	scalefree	0.15	0.225	0.098	0.218	0.096	0.064	0.049	0.136
SIS	scalefree	0.20	0.226	0.098	0.222	0.09	0.052	0.04	0.135
SIS	smallworld	0.10	0.018	0.075	0.045	−0.02	−0.014	0.076	−0.014
SIS	smallworld	0.15	0.09	0.261	0.16	0.054	0.068	0.221	0.056
SIS	smallworld	0.20	0.131	0.236	0.239	0.095	0.146	0.255	0.136

Top Ten Overlap of Centrality Measures and Maximum Delta TKO on Base Network

Disease Type	Network Type	InfectionRate	Degree	Closeness	Betweenness	Eigenvector	Katz	Accessibility	Expected Force
SIR	scalefree	0.10	0.08	0.092	0.076	0.08	0.076	0.08	0.092
SIR	scalefree	0.15	0.124	0.132	0.132	0.124	0.116	0.128	0.128
SIR	scalefree	0.20	0.1	0.092	0.108	0.088	0.092	0.092	0.096
SIR	smallworld	0.10	0.064	0.072	0.064	0.024	0.048	0.088	0.068
SIR	smallworld	0.15	0.12	0.128	0.128	0.08	0.096	0.108	0.096
SIR	smallworld	0.20	0.112	0.128	0.128	0.056	0.104	0.108	0.112
SIS	scalefree	0.10	0.12	0.1	0.12	0.1	0.104	0.112	0.124
SIS	scalefree	0.15	0.116	0.108	0.108	0.104	0.092	0.096	0.108
SIS	scalefree	0.20	0.092	0.088	0.092	0.068	0.068	0.08	0.088
SIS	smallworld	0.10	0.088	0.088	0.092	0.068	0.084	0.08	0.096
SIS	smallworld	0.15	0.1	0.116	0.108	0.064	0.104	0.16	0.096
SIS	smallworld	0.20	0.14	0.152	0.172	0.044	0.164	0.164	0.164

Table 8. The Pearson and Spearman correlations as well as the average percent of matching Top Ten agents between the maximum change in fractional TKO score with each of seven base network agent centrality scores.

Maximum Delta Fraction TKO and Flattened Interaction Network - Unweighted

Pearson Correlations of Unweighted Centrality Measures and Maximum Delta TKO on Flattened Network

Disease Type	Network Type	InfectionRate	Degree	Closeness	Betweenness	Eigenvector	Katz	Accessibility	Expected Force
SIR	scalefree	0.10	0.103	0.081	0.081	0.05	−0.01	0.065	0.094
SIR	scalefree	0.15	0.166	0.129	0.151	0.062	0.026	0.109	0.149
SIR	scalefree	0.20	0.154	0.122	0.129	0.042	0.01	0.099	0.141
SIR	smallworld	0.10	0.005	0.087	0.047	−0.037	−0.003	0.074	0.001
SIR	smallworld	0.15	0.086	0.184	0.147	0.079	0.002	0.198	0.086
SIR	smallworld	0.20	0.111	0.207	0.168	−0.004	−0.005	0.203	0.109
SIS	scalefree	0.10	0.118	0.082	0.102	0.066	0.009	0.068	0.099
SIS	scalefree	0.15	0.138	0.096	0.116	0.084	0.025	0.071	0.118
SIS	scalefree	0.20	0.115	0.09	0.095	0.036	0.019	0.066	0.103
SIS	smallworld	0.10	0.018	0.116	0.045	−0.02	0.004	0.079	0.014
SIS	smallworld	0.15	0.081	0.236	0.158	0.039	0.015	0.21	0.079
SIS	smallworld	0.20	0.141	0.201	0.269	−0.044	0.014	0.26	0.142

Spearman Correlations of Unweighted Centrality Measures and Maximum Delta TKO on Flattened Network

Disease Type	Network Type	InfectionRate	Degree	Closeness	Betweenness	Eigenvector	Katz	Accessibility	Expected Force
SIR	scalefree	0.10	0.192	0.104	0.191	0.065	−0.023	0.071	0.13
SIR	scalefree	0.15	0.222	0.122	0.217	0.074	0.018	0.088	0.155
SIR	scalefree	0.20	0.232	0.135	0.225	0.053	0.007	0.097	0.166
SIR	smallworld	0.10	0.016	0.055	0.049	−0.06	0.006	0.069	−0.002
SIR	smallworld	0.15	0.061	0.177	0.097	0.113	0.005	0.155	0.052
SIR	smallworld	0.20	0.099	0.241	0.141	0.029	0.01	0.204	0.071
SIS	scalefree	0.10	0.184	0.088	0.188	0.052	0.004	0.054	0.115
SIS	scalefree	0.15	0.225	0.096	0.221	0.076	0.038	0.049	0.136
SIS	scalefree	0.20	0.226	0.098	0.221	0.043	0.025	0.041	0.136
SIS	smallworld	0.10	0.018	0.075	0.045	−0.015	−0.012	0.076	−0.014
SIS	smallworld	0.15	0.09	0.261	0.16	0.108	0.01	0.221	0.056
SIS	smallworld	0.20	0.131	0.236	0.239	0.007	−0.016	0.255	0.136

Top Ten Overlap of Unweighted Centrality Measures and Maximum Delta TKO on Flattened Network

Disease Type	Network Type	InfectionRate	Degree	Closeness	Betweenness	Eigenvector	Katz	Accessibility	Expected Force
SIR	scalefree	0.10	0.084	0.088	0.076	0.092	0.052	0.092	0.092
SIR	scalefree	0.15	0.124	0.128	0.132	0.068	0.108	0.128	0.128
SIR	scalefree	0.20	0.1	0.088	0.108	0.04	0.092	0.088	0.096
SIR	smallworld	0.10	0.064	0.072	0.064	0.052	0.028	0.088	0.068
SIR	smallworld	0.15	0.12	0.128	0.128	0.076	0.04	0.112	0.096
SIR	smallworld	0.20	0.112	0.128	0.128	0.072	0.036	0.108	0.112
SIS	scalefree	0.10	0.128	0.1	0.12	0.112	0.052	0.108	0.124
SIS	scalefree	0.15	0.108	0.092	0.108	0.08	0.076	0.088	0.104
SIS	scalefree	0.20	0.092	0.088	0.096	0.056	0.084	0.08	0.088
SIS	smallworld	0.10	0.088	0.088	0.092	0.072	0.048	0.08	0.096
SIS	smallworld	0.15	0.1	0.116	0.108	0.048	0.056	0.164	0.096
SIS	smallworld	0.20	0.14	0.152	0.172	0.04	0.068	0.164	0.164

Table 9. The Pearson and Spearman correlations as well as the average percent of matching Top Ten agents between the maximum change in fractional TKO score with each of seven flattened observed interaction network agent centrality scores.

Mean TKO and Base Interaction Network

Pearson Correlations of Centrality Measures and Mean TKO on Base Network

Disease Type	Network Type	InfectionRate	Degree	Closeness	Betweenness	Eigenvector	Katz	Accessibility	Expected Force
SIR	scalefree	0.10	0.127	0.106	0.099	0.098	0.08	0.081	0.119
SIR	scalefree	0.15	0.125	0.096	0.102	0.098	0.084	0.069	0.114
SIR	scalefree	0.20	0.14	0.119	0.115	0.115	0.101	0.096	0.133
SIR	smallworld	0.10	−0.005	0.031	0.019	−0.008	−0.015	0.039	−0.006
SIR	smallworld	0.15	0.049	0.07	0.082	0.08	0.064	0.091	0.051
SIR	smallworld	0.20	0.067	0.127	0.102	0.026	0.057	0.136	0.062
SIS	scalefree	0.10	0.135	0.089	0.111	0.091	0.068	0.061	0.111
SIS	scalefree	0.15	0.162	0.098	0.127	0.106	0.085	0.054	0.13
SIS	scalefree	0.20	0.233	0.167	0.191	0.168	0.138	0.107	0.201
SIS	smallworld	0.10	0.025	0.054	0.02	−0.013	0.004	0.041	0.022
SIS	smallworld	0.15	0.068	0.102	0.11	0.025	0.056	0.107	0.066
SIS	smallworld	0.20	0.123	0.174	0.241	0.052	0.126	0.232	0.121

Spearman Correlations of Centrality Measures and Mean TKO on Base Network

Disease Type	Network Type	InfectionRate	Degree	Closeness	Betweenness	Eigenvector	Katz	Accessibility	Expected Force
SIR	scalefree	0.10	0.191	0.092	0.191	0.081	0.047	0.051	0.12
SIR	scalefree	0.15	0.21	0.112	0.19	0.108	0.077	0.069	0.141
SIR	scalefree	0.20	0.222	0.131	0.215	0.123	0.094	0.085	0.155
SIR	smallworld	0.10	0.006	0.015	0.025	0.025	0.007	0.031	−0.001
SIR	smallworld	0.15	0.052	0.088	0.083	0.07	0.054	0.088	0.045
SIR	smallworld	0.20	0.056	0.157	0.096	0.056	0.058	0.131	0.047
SIS	scalefree	0.10	0.2	0.084	0.203	0.074	0.039	0.036	0.115
SIS	scalefree	0.15	0.277	0.1	0.263	0.099	0.054	0.029	0.151
SIS	scalefree	0.20	0.321	0.136	0.314	0.126	0.073	0.043	0.187
SIS	smallworld	0.10	0.008	0.024	0.019	−0.014	−0.017	0.036	−0.019
SIS	smallworld	0.15	0.067	0.159	0.134	0.036	0.048	0.142	0.044
SIS	smallworld	0.20	0.115	0.203	0.217	0.07	0.116	0.219	0.102

Top Ten Overlap of Centrality Measures and Mean TKO on Base Network

Disease Type	Network Type	InfectionRate	Degree	Closeness	Betweenness	Eigenvector	Katz	Accessibility	Expected Force
SIR	scalefree	0.10	0.108	0.12	0.096	0.108	0.112	0.108	0.116
SIR	scalefree	0.15	0.112	0.116	0.112	0.116	0.116	0.12	0.112
SIR	scalefree	0.20	0.108	0.112	0.116	0.112	0.116	0.1	0.104
SIR	smallworld	0.10	0.04	0.06	0.044	0.044	0.032	0.08	0.052
SIR	smallworld	0.15	0.096	0.12	0.108	0.088	0.096	0.104	0.084
SIR	smallworld	0.20	0.124	0.128	0.1	0.056	0.112	0.092	0.108
SIS	scalefree	0.10	0.12	0.112	0.108	0.108	0.108	0.116	0.124
SIS	scalefree	0.15	0.108	0.088	0.1	0.084	0.084	0.084	0.092
SIS	scalefree	0.20	0.144	0.128	0.136	0.12	0.128	0.116	0.136
SIS	smallworld	0.10	0.076	0.056	0.072	0.056	0.06	0.052	0.08
SIS	smallworld	0.15	0.12	0.132	0.12	0.064	0.1	0.124	0.096
SIS	smallworld	0.20	0.172	0.156	0.196	0.056	0.184	0.156	0.184

Table 10. The Pearson and Spearman correlations as well as the average percent of matching Top Ten agents between the mean proportional TKO score with each of seven base network agent centrality scores.

Mean TKO and Flattened Interaction Network - Unweighted

Pearson Correlations of Unweighted Centrality Measures and Mean TKO on Flattened Network

Disease Type	Network Type	InfectionRate	Degree	Closeness	Betweenness	Eigenvector	Katz	Accessibility	Expected Force
SIR	scalefree	0.10	0.128	0.104	0.101	0.06	−0.015	0.081	0.119
SIR	scalefree	0.15	0.126	0.095	0.104	0.061	−0.01	0.069	0.114
SIR	scalefree	0.20	0.14	0.119	0.116	0.045	0.014	0.096	0.134
SIR	smallworld	0.10	−0.005	0.031	0.019	−0.032	−0.013	0.039	−0.006
SIR	smallworld	0.15	0.049	0.07	0.082	0.047	−0.006	0.091	0.051
SIR	smallworld	0.20	0.067	0.127	0.102	−0.007	0.016	0.136	0.062
SIS	scalefree	0.10	0.136	0.087	0.113	0.08	0.013	0.061	0.111
SIS	scalefree	0.15	0.162	0.095	0.128	0.099	0.017	0.053	0.13
SIS	scalefree	0.20	0.234	0.168	0.193	0.107	0.016	0.107	0.202
SIS	smallworld	0.10	0.025	0.054	0.02	0.008	0.006	0.041	0.022
SIS	smallworld	0.15	0.068	0.102	0.11	0.024	0.015	0.107	0.066
SIS	smallworld	0.20	0.123	0.174	0.241	0.001	−0.004	0.232	0.121

Spearman Correlations of Unweighted Centrality Measures and Mean TKO on Flattened Network

Disease Type	Network Type	InfectionRate	Degree	Closeness	Betweenness	Eigenvector	Katz	Accessibility	Expected Force
SIR	scalefree	0.10	0.19	0.092	0.192	0.053	−0.018	0.051	0.12
SIR	scalefree	0.15	0.21	0.112	0.193	0.072	0.029	0.071	0.142
SIR	scalefree	0.20	0.222	0.131	0.214	0.043	−0.003	0.086	0.156
SIR	smallworld	0.10	0.006	0.015	0.025	−0.043	0.002	0.031	−0.001
SIR	smallworld	0.15	0.052	0.088	0.083	0.053	0.008	0.088	0.045
SIR	smallworld	0.20	0.056	0.157	0.096	0.03	0.009	0.131	0.047
SIS	scalefree	0.10	0.2	0.083	0.204	0.053	0.006	0.036	0.115
SIS	scalefree	0.15	0.277	0.098	0.265	0.079	0.046	0.029	0.152
SIS	scalefree	0.20	0.321	0.136	0.313	0.054	0.023	0.044	0.189
SIS	smallworld	0.10	0.008	0.024	0.019	−0.01	−0.009	0.036	−0.019
SIS	smallworld	0.15	0.067	0.159	0.134	0.06	0.007	0.143	0.044
SIS	smallworld	0.20	0.115	0.203	0.217	0.023	−0.009	0.219	0.102

Top Ten Overlap of Unweighted Centrality Measures and Mean TKO on Flattened Network

Disease Type	Network Type	InfectionRate	Degree	Closeness	Betweenness	Eigenvector	Katz	Accessibility	Expected Force
SIR	scalefree	0.10	0.104	0.112	0.096	0.092	0.036	0.116	0.116
SIR	scalefree	0.15	0.112	0.112	0.112	0.08	0.096	0.116	0.112
SIR	scalefree	0.20	0.108	0.108	0.116	0.076	0.072	0.096	0.104
SIR	smallworld	0.10	0.04	0.06	0.044	0.052	0.036	0.08	0.052
SIR	smallworld	0.15	0.096	0.12	0.108	0.04	0.036	0.1	0.084
SIR	smallworld	0.20	0.124	0.128	0.1	0.056	0.064	0.092	0.108
SIS	scalefree	0.10	0.124	0.108	0.112	0.088	0.06	0.116	0.124
SIS	scalefree	0.15	0.108	0.076	0.1	0.084	0.072	0.072	0.088
SIS	scalefree	0.20	0.144	0.128	0.14	0.128	0.076	0.12	0.136
SIS	smallworld	0.10	0.076	0.056	0.072	0.08	0.032	0.052	0.08
SIS	smallworld	0.15	0.12	0.132	0.12	0.048	0.052	0.124	0.096
SIS	smallworld	0.20	0.172	0.156	0.196	0.044	0.048	0.156	0.184

Table 11. The Pearson and Spearman correlations as well as the average percent of matching Top Ten agents between the mean TKO score with each of seven flattened observed interaction network agent centrality scores.

Mean Delta Fraction TKO and Base Interaction Network

Pearson Correlations of Centrality Measures and Mean Delta TKO on Base Network

Disease Type	Network Type	InfectionRate	Degree	Closeness	Betweenness	Eigenvector	Katz	Accessibility	Expected Force
SIR	scalefree	0.10	0.111	0.098	0.088	0.088	0.072	0.078	0.106
SIR	scalefree	0.15	0.149	0.117	0.13	0.124	0.111	0.095	0.136
SIR	scalefree	0.20	0.168	0.146	0.14	0.142	0.128	0.12	0.161
SIR	smallworld	0.10	0.	0.048	0.029	−0.013	−0.015	0.053	−0.002
SIR	smallworld	0.15	0.076	0.156	0.12	0.08	0.085	0.169	0.077
SIR	smallworld	0.20	0.104	0.179	0.141	0.037	0.09	0.183	0.099
SIS	scalefree	0.10	0.126	0.085	0.105	0.089	0.069	0.063	0.104
SIS	scalefree	0.15	0.183	0.117	0.146	0.125	0.102	0.072	0.152
SIS	scalefree	0.20	0.245	0.176	0.201	0.177	0.147	0.113	0.212
SIS	smallworld	0.10	0.024	0.084	0.03	−0.012	0.001	0.059	0.019
SIS	smallworld	0.15	0.092	0.221	0.152	0.043	0.079	0.191	0.089
SIS	smallworld	0.20	0.148	0.243	0.282	0.068	0.151	0.285	0.146

Spearman Correlations of Centrality Measures and Mean Delta TKO on Base Network

Disease Type	Network Type	InfectionRate	Degree	Closeness	Betweenness	Eigenvector	Katz	Accessibility	Expected Force
SIR	scalefree	0.10	0.19	0.096	0.191	0.084	0.049	0.056	0.122
SIR	scalefree	0.15	0.218	0.12	0.208	0.117	0.088	0.083	0.151
SIR	scalefree	0.20	0.227	0.138	0.219	0.129	0.1	0.094	0.162
SIR	smallworld	0.10	0.01	0.039	0.039	0.026	0.006	0.053	−0.003
SIR	smallworld	0.15	0.052	0.146	0.081	0.079	0.06	0.132	0.048
SIR	smallworld	0.20	0.07	0.212	0.122	0.057	0.065	0.173	0.054
SIS	scalefree	0.10	0.198	0.087	0.202	0.077	0.042	0.04	0.116
SIS	scalefree	0.15	0.292	0.109	0.283	0.109	0.063	0.037	0.164
SIS	scalefree	0.20	0.327	0.138	0.321	0.128	0.073	0.044	0.191
SIS	smallworld	0.10	0.009	0.053	0.035	−0.025	−0.023	0.056	−0.024
SIS	smallworld	0.15	0.072	0.248	0.14	0.055	0.059	0.205	0.046
SIS	smallworld	0.20	0.118	0.256	0.227	0.076	0.12	0.244	0.104

Top Ten Overlap of Centrality Measures and Mean Delta TKO on Base Network

Disease Type	Network Type	InfectionRate	Degree	Closeness	Betweenness	Eigenvector	Katz	Accessibility	Expected Force
SIR	scalefree	0.10	0.108	0.116	0.096	0.108	0.112	0.108	0.112
SIR	scalefree	0.15	0.136	0.14	0.144	0.14	0.14	0.14	0.136
SIR	scalefree	0.20	0.132	0.124	0.132	0.124	0.132	0.112	0.124
SIR	smallworld	0.10	0.052	0.064	0.056	0.044	0.036	0.096	0.056
SIR	smallworld	0.15	0.108	0.128	0.128	0.088	0.1	0.12	0.096
SIR	smallworld	0.20	0.144	0.14	0.128	0.06	0.124	0.108	0.12
SIS	scalefree	0.10	0.136	0.128	0.128	0.124	0.12	0.128	0.136
SIS	scalefree	0.15	0.128	0.108	0.116	0.104	0.104	0.104	0.112
SIS	scalefree	0.20	0.144	0.128	0.14	0.12	0.128	0.12	0.14
SIS	smallworld	0.10	0.08	0.072	0.08	0.076	0.068	0.048	0.088
SIS	smallworld	0.15	0.128	0.16	0.136	0.076	0.132	0.168	0.124
SIS	smallworld	0.20	0.184	0.168	0.204	0.068	0.212	0.18	0.2

Table 12. The Pearson and Spearman correlations as well as the average percent of matching Top Ten agents between the mean change in fractional TKO score with each of seven base network agent centrality scores.

Mean Delta Fraction TKO and Flattened Interaction Network - Unweighted

Pearson Correlations of Unweighted Centrality Measures and Mean Delta TKO on Flattened Network

Disease Type	Network Type	InfectionRate	Degree	Closeness	Betweenness	Eigenvector	Katz	Accessibility	Expected Force
SIR	scalefree	0.10	0.112	0.097	0.09	0.046	−0.01	0.077	0.106
SIR	scalefree	0.15	0.149	0.116	0.131	0.073	0.004	0.094	0.136
SIR	scalefree	0.20	0.168	0.146	0.14	0.058	0.012	0.121	0.162
SIR	smallworld	0.10	0.	0.048	0.029	−0.035	−0.009	0.053	−0.002
SIR	smallworld	0.15	0.076	0.156	0.12	0.061	−0.002	0.169	0.077
SIR	smallworld	0.20	0.104	0.179	0.141	0.	0.003	0.183	0.099
SIS	scalefree	0.10	0.127	0.083	0.106	0.076	0.015	0.062	0.104
SIS	scalefree	0.15	0.183	0.113	0.147	0.116	0.021	0.07	0.152
SIS	scalefree	0.20	0.246	0.176	0.203	0.114	0.018	0.113	0.212
SIS	smallworld	0.10	0.024	0.084	0.03	−0.006	0.012	0.059	0.019
SIS	smallworld	0.15	0.092	0.221	0.152	0.041	0.013	0.191	0.089
SIS	smallworld	0.20	0.148	0.243	0.282	−0.006	0.	0.285	0.146

Spearman Correlations of Unweighted Centrality Measures and Mean Delta TKO on Flattened Network

Disease Type	Network Type	InfectionRate	Degree	Closeness	Betweenness	Eigenvector	Katz	Accessibility	Expected Force
SIR	scalefree	0.10	0.19	0.096	0.191	0.051	−0.024	0.057	0.122
SIR	scalefree	0.15	0.218	0.12	0.21	0.068	0.022	0.084	0.151
SIR	scalefree	0.20	0.227	0.139	0.218	0.048	0.003	0.095	0.163
SIR	smallworld	0.10	0.01	0.039	0.039	−0.052	0.007	0.053	−0.003
SIR	smallworld	0.15	0.052	0.146	0.081	0.092	0.013	0.132	0.048
SIR	smallworld	0.20	0.07	0.212	0.122	0.032	0.007	0.173	0.054
SIS	scalefree	0.10	0.198	0.086	0.203	0.052	0.012	0.041	0.117
SIS	scalefree	0.15	0.292	0.107	0.285	0.088	0.046	0.037	0.164
SIS	scalefree	0.20	0.327	0.138	0.32	0.059	0.024	0.045	0.192
SIS	smallworld	0.10	0.009	0.053	0.035	−0.01	−0.008	0.055	−0.024
SIS	smallworld	0.15	0.072	0.248	0.14	0.112	0.009	0.205	0.046
SIS	smallworld	0.20	0.118	0.256	0.227	0.017	−0.018	0.243	0.104

Top Ten Overlap of Unweighted Centrality Measures and Mean Delta TKO on Flattened Network

Disease Type	Network Type	InfectionRate	Degree	Closeness	Betweenness	Eigenvector	Katz	Accessibility	Expected Force
SIR	scalefree	0.10	0.108	0.116	0.1	0.072	0.04	0.116	0.112
SIR	scalefree	0.15	0.132	0.132	0.144	0.096	0.104	0.132	0.132
SIR	scalefree	0.20	0.132	0.12	0.132	0.088	0.076	0.108	0.124
SIR	smallworld	0.10	0.052	0.064	0.056	0.052	0.048	0.096	0.056
SIR	smallworld	0.15	0.108	0.128	0.128	0.06	0.048	0.116	0.096
SIR	smallworld	0.20	0.144	0.14	0.128	0.064	0.04	0.108	0.12
SIS	scalefree	0.10	0.14	0.12	0.132	0.092	0.068	0.128	0.136
SIS	scalefree	0.15	0.124	0.092	0.116	0.092	0.076	0.088	0.104
SIS	scalefree	0.20	0.144	0.128	0.144	0.128	0.084	0.124	0.14
SIS	smallworld	0.10	0.08	0.072	0.08	0.084	0.052	0.048	0.088
SIS	smallworld	0.15	0.128	0.16	0.136	0.048	0.048	0.168	0.124
SIS	smallworld	0.20	0.184	0.168	0.204	0.04	0.04	0.18	0.2

Table 13. The Pearson and Spearman correlations as well as the average percent of matching Top Ten agents between the mean change in fractional TKO score with each of seven flattened observed interaction network agent centrality scores.
